# DNA Phosphorothioate Modification Plays a Role in Peroxides Resistance in *Streptomyces lividans*

**DOI:** 10.3389/fmicb.2016.01380

**Published:** 2016-08-31

**Authors:** Daofeng Dai, Aiqin Du, Kangli Xiong, Tianning Pu, Xiufen Zhou, Zixin Deng, Jingdan Liang, Xinyi He, Zhijun Wang

**Affiliations:** State Key Laboratory of Microbial Metabolism and School of Life Science and Biotechnology, Shanghai Jiao Tong UniversityShanghai, China

**Keywords:** DNA phosphorothioate modification, organic hydroperoxide resistance, antioxidant, catalase

## Abstract

DNA phosphorothioation, conferred by *dnd* genes, was originally discovered in the soil-dwelling bacterium *Streptomyces lividans*, and thereafter found to exist in various bacterial genera. However, the physiological significance of this sulfur modification of the DNA backbone remains unknown in *S. lividans*. Our studies indicate that DNA phosphorothioation has a major role in resistance to oxidative stress in the strain. Although *Streptomyces* species express multiple catalase/peroxidase and organic hydroperoxide resistance genes to protect them against peroxide damage, a wild type strain of *S. lividans* exhibited two-fold to 10-fold higher survival, compared to a *dnd*^−^ mutant, following treatment with peroxides. RNA-seq experiments revealed that, catalase and organic hydroperoxide resistance gene expression were not up-regulated in the wild type strain, suggesting that the resistance to oxidative stress was not due to the up-regulation of these genes by DNA phosphorothioation. Quantitative RT-PCR analysis was conducted to trace the expression of the catalase and the organic hydroperoxide resistance genes after peroxides treatments. A bunch of these genes were activated in the *dnd*^−^ mutant rather than the wild type strain in response to peroxides. Moreover, the organic hydroperoxide peracetic acid was scavenged more rapidly in the presence than in the absence of phosphorothioate modification, both *in vivo* and *in vitro*. The *dnd* gene cluster can be up-regulated by the disulfide stressor diamide. Overall, our observations suggest that DNA phosphorothioate modification functions as a peroxide resistance system in *S. lividans*.

## Introduction

DNA phosphorothioate modification, in which a non-bridging oxygen in DNA phosphate backbone is replaced by sulfur, exists widely in bacteria. Originally, the modification was found in the soil-inhabiting, antibiotic-producing *Streptomyces* species. Through the DND (DNA degradation) phenotype and *in silico* analysis, the modification was also found to distribute in human pathogens, including *Mycobacterium abscessus*, an emerging respiratory pathogen of cystic fibrosis patients, *Clostridium difficile*, the main etiologic agent of nosocomial diarrhea, and *Salmonella enterica*, a cause of intestinal inflammation (He et al., 2007; Wang et al., 2007, 2011; Ou et al., 2009).

Phosphorothioate modification is stereo-specific, with only the R configuration found in bacteria (Wang et al., 2007), and it is also sequence-selective (Boybek et al., 1998; Dyson and Evans, 1998; Zhou et al., 2005; Liang et al., 2007; Wang et al., 2011; Cao et al., 2014a). Quantitative analysis shows that three discrete genomic modification frequencies, ranging from two to three modifications per 10^3^ nt, three to eight per 10^4^ nt, and two to six hundreds per 10^6^ nt, along with a well-conserved target sequence of three to five nucleotides in length, exist in different bacterial species (Wang et al., 2011). Although the modification nucleotide sequence is well conserved, only a fraction of the potential sites are modified. In a plasmid from *S. lividans* 1326 containing 14 potential modification sites, DNA phosphorothioation occurred selectively once per plasmid (Liang et al., 2007). The partial modification was further confirmed in plasmid and genomic DNA from *Escherichia coli* B7A, and estimated to occupy 12–14% of all potential sites (Cao et al., 2014a).

DNA phosphorothioation is determined by the products of a five-membered gene cluster termed *dnd* (Zhou et al., 2005). Four of the genes, *dndA* and *dndCDE* are essential for the modification. The product of *dndB* was shown to suppress the expression of dnd gene cluster (Liang et al., 2007; Cheng et al., 2015; He et al., 2015).

Studies with chemically phosphorothioated DNA revealed that the modification confers resistance to nuclease digestion (Eckstein, 2000), suggesting that the modification might be part of a host restriction system (Wang et al., 2007). Indeed, a decrease in transformation rate was observed, comparing between a phosphorothioate-modified plasmid and its non-modified counterpart in *Salmonella enterica* (Xu et al., 2010). A DNA damaging phenotype was also seen when the modification gene cluster was knocked out, and several genes downstream of the *dnd* genes were involved in the process (Cao et al., 2014a,b; Gan et al., 2014). Interestingly, phosphorothioated DNA from *S. lividans* was specifically recognized by a restriction endonuclease in its sister species *S. coelicolor*. NMR structural studies demonstrate that the sulfur in [Rp, Rp]-phosphorothioate dsDNA locates in the major groove facilitating selective interactions with the restriction endonuclease (Liu et al., 2015; Lan et al., 2016). As with many other species, the *dnd* genes of *S. lividans* are located on a genomic island, which is absent from *S. coelicolor* (Liu et al., 2010; Howard et al., 2013).

We showed previously that phosphorothioated DNA has reducing properties, reacting with peroxides stoichiometrically (Xie et al., 2012). Growth curve analyses with *Salmonella enterica* indicated that DNA phosphorothioation provided a survival advantage during exposure to oxidative stress. Using a very high concentration of hydrogen peroxide (880 mM) to treat the cells, phosphorothioate modification in DNA was also found to be consumed. It was therefore suggested that the phosphorothioate modification function as an anti-oxidant in the bacteria (Xie et al., 2012). *Salmonella* spp. is a facultative intracellular bacterium that causes a wide range of infections in mammals. The ability of the pathogen to survive and proliferate within host cells relies on its arsenal of detoxifying enzymes to cope with Phox-mediated oxidative stress (Aussel et al., 2011). Additional, anti-oxidation capability such as DNA phosphorothioation might not be redundant. However, *Streptomyces* spp. is known to harbor multiple catalase and organic hydroperoxide resistance genes (Cruz-Morales et al., 2013), it is intriguing whether the modification still function as an anti-oxidation system in the species.

The chemical results of phosphorothioate reaction with peroxides are different. After oxidized by hydrogen peroxide, the majority of phosphorothioate bonds were converted to normal DNA backbone, which should not be harmful to the bacteria if this happens *in vivo*. However, the phosphorothioate bond can be efficiently cleaved by peracetic acid (PAA), resulting degraded DNA fragments (Ray et al., 1995; Liang et al., 2007; Xie et al., 2012). Therefore, it is important to understand whether the phosphorothioate DNA modification alleviates or augments oxidative stress exerted particularly by PAA on the host.

In addition, the modification frequency of two to seven hundreds per 10^6^ nt, which equals to around 10 μM per cell, appears too low for stoichiometric counteraction against peroxides at millimolar concentration. This raises a possibility that there may be broader interactions between phosphorothioate DNA and the physiological response to oxidative stress. For example, phosphorothioation may function as a regulatory switch that controls the expression of genes involved in resistance to oxidation. Indeed, effects of phosphorothioation on DNA include destabilizing the B-type helix, and enhancing the stiffness of the backbone through interactions with the base-pair steps (Zhang et al., 2012; Chen et al., 2015), which could potentially alter the interaction of transcriptional factors with the modified DNA, such as those observed in DNA methylation (Marinus and Casadesus, 2009).

Although many enzymes and small molecules can scavenge H_2_O_2_ *in vitro*, they may not serve as the same function *in vivo*. It was therefore suggested that several aspects should be examined before coming to the conclusion of their anti-oxidation functions. These aspects include a characteristic sensitivity to peroxide if the related gene is absent, catalytic efficiency of the small molecules or enzymes, and the induction of the gene by an oxidant (Mishra and Imlay, 2012).

We sought to address these questions in this study, and show that DNA phosphorothioate modification is an anti-oxidation system in *S. lividans* that preferentially scavenges organic hydroperoxides, and that the *dnd* gene cluster is subjected to diamide regulation.

## Materials and methods

### Strains, plasmids, and media

Bacterial strains and plasmids used in this work are listed in Table S1. *Streptomyces* strains were cultured in rich liquid media TSBY (30 g/L tryptic soy broth, 5 g/L yeast, 340 g/L sucrose) or minimal M9 media (6.8 g/L Na_2_HPO_4_, 3.0 g/L KH_2_PO_4_, 0.5 g/L NaCl, 1.0 g/L NH_4_Cl) supplemented with 5 g/L w/v glucose and 0.12 g/L MgSO_4_. Antibiotics were added to the medium as needed (50 μg/ml for apramycin).

To generate the reporter vector P*dndB*-*xylE*, which contains *xylE* under the control of the *dndB* promoter, a DNA fragment that included the *xylE* coding region, ribosomal binding site (RBS), and upstream region containing stop codons in all three reading frames was amplified from pIJ4083 (Tunca et al., 2007) by PCR with primers 3700F and 3700R (Table S2). The PCR product was digested using *Xba*I, and ligated into the *Xba*I-digested pSET152, generating the *xylE* reporter vector, pJTU3700. Next, a 760-bp fragment containing the intergenic region between *dndA* and *dndB* (from positions −387 to 373 with respect to the *dndB* transcriptional start site, determined in this study) was amplified from chromosomal DNA of *S. lividans* 1326 by PCR with primer PdndBF and PdndBR (Table S2). The PCR product was digested using *Bam*HI, and ligated with *Bam*HI-digested plasmid pJTU3700, producing the *PdndB*-*xylE* reporter plasmid pJTU3707. The reporter pJTU3707 was then introduced into *S. lividans* 1326 via conjugation using *E. coli* ET12567 (pUZ8002). The catechol-2,3-dioxygenase activity assay was performed as described without modification (Ingram et al., 1989).

To generate plasmid pJTU3707-10, the -10 region of the *dndB* promoter was removed from pJTU3707 using PCR. Briefly, after PCR amplification using pJTU3707 as the template, 1 μl of *Dpn*I was added to the PCR reaction mixture and incubated at 37°C for 6 h to remove the template plasmid. The PCR product was transformed into *E. coli* DH5α, resulting in plasmid pJTU3707-10.

Disruption of *sigR* was performed by gene replacement via homologous recombination. For this purpose, two DNA fragments containing partial *sigR* sequence and its flanking region were amplified from *S. lividans* 1326 genomic DNA by PCR using KOD plus DNA polymerase (TOYOBO, Japan). A 1582 bp PCR product (primer 5505LF and 5505LR), and a 1552 bp PCR (primer 5505RF and 5505RR) were digested with XbaI and BglII, and EcoRI and BglII respectively. The plasmid pBluescript II was digested using XbaI and EcoRI. Then the PCR DNA fragments were inserted into pBluescript II, generating pJTU3730. The cassette containing apramycin-resistance gene and OriT was obtained from pIJ773 by PCR amplification using primer 773F and 773R (Table S2). The BglII-digested cassette from pIJ773 was inserted into BglII-digested pJTU3730, generating pJTU3731, which pJTU3731 was then introduced into *S. lividans* 1326 via conjugation mediated by *E. coli* ET12567 (pUZ8002) (Kieser et al., 2000). The resulting exconjugants were cultured on MS plates. Spores were harvested and spread again on MS plates containing apramycin. After culture at 30°C for 3 days, apramycin resistance (*apr*^*r*^) colonies were selected and screened for *sigR* disruption mutants by PCR (using primer 5505TF and 5505TR).

### Survival assay

To test survival following exposure to oxidative agents, strains of *S. lividans* 1326 and HXY6 containing the plasmid pIJ702 were used (Zhu et al., 2005). Fresh spores were pre-germinated according to the standard protocol without modification (Kieser et al., 2000). Briefly, stock spores were pelleted by centrifugation and re-suspended in TES buffer (0.05 M, pH8.0). The spores were heat shocked at 50°C for 10 min, cooled using cold tap water, and then an equal volume of double-strength Germination Medium (1% Difco yeast extract, 1% Difco casaminoacids, and 0.01 M CaCl_2_) was added. The spores were then incubated at 37°C with shaking at 300 rpm for 2–3 h. Approximately 1 × 10^7^ germinated spores (calibrated by spectrophotometry using OD_450_) were washed twice using distilled water, and then diluted 100-fold prior to peroxide treatment. For the H_2_O_2_ treatment, H_2_O_2_ was added to a final concentration of 20 mM, and spores were incubated at 30°C for 2 h. Samples were taken at 30 min intervals, and diluted 100-fold using distilled water. PAA was added to final concentrations of 200, 230, 260, and 290 μM, and spores were incubated at 30°C for 30 min. Cumene hydroperoxide (CHP) was added to final concentrations of 10, 12.5, 15, 17.5, and 20 mM, and spores were incubated at 30°C for 1 h. Diamide was added to final concentrations of 15, 30, and 45 mM, and spores were incubated at 30°C for 1 h. Controls consisted of samples not exposed to oxidants. Aliquots were plated on MMT media, incubated for 4 days at 30°C, and the number of viable cells was determined by colony counts.

### *In vivo* and *in vitro* scavenging of PAA

Spores of *S. lividans* 1326 and HXY6 were inoculated into TSBY media. Following growth for 3 days at 30°C with shaking at 220 rpm, mycelia were collected by centrifugation. To measure *in vivo* PAA-scavenging, mycelia were washed twice using M9 media and re-suspended in the same media to an OD_450_ of 0.2. After growing for 3 h, cultures were challenged with 500 μM PAA, incubated at 30°C with shaking for 3 h, and then samples were withdrawn at 30 min intervals. The concentration of residual PAA was measured in 96-well plates using Gen5 (Biotek) with the Hydrogen Peroxide Quantitative Assay Kit (Sangon Biotech), which is based on the ferric-xylenol orange assay and which can detect peroxides in the micromolar concentration range (Gay and Gebicki, 2002).

For the *in vitro* PAA-scavenging assay, mycelia were washed twice and re-suspended in buffer containing 50 mM potassium phosphate (pH 7.6) (P buffer). Cells were disrupted by sonication and then centrifuged at 15000 rpm for 20 min at 4°C to remove cell debris. Protein concentration was measured using the Bradford Protein Assay, and the protein concentrations of different strains were adjusted to the same level by dilution with P buffer. The total protein extracts were treated with 5 mM PAA for 2.5 h. Samples were then taken at 5 min intervals, and the PAA concentration was measured as described above.

### RT-PCR and quantitative RT-PCR

*S. lividans* 1326 was cultured in TSBY medium to an OD_450_ reading of 0.4, and then treated with 1 mM paraquat, 20 mM H_2_O_2_, 200 μM PAA, 3 mM CHP, or 3 mM diamide. Samples were collected by centrifugation at 0, 25, 50, or 75 min post addition of oxidants to the culture. For the analysis of peroxide scavenging genes, samples were collected at 15 min intervals. RNA was then isolated and RNA concentration was measured using the Nanodrop 2000 Spectrophotometer (Thermo Fisher Scientific Inc.). Genomic DNA was removed using RNase-free DNase I, and the absence of DNA contamination was further confirmed using PCR. Reverse transcription was performed using the cDNA Synthesis Kit (Thermo Fisher Scientific Inc.), and the resulting cDNA was used as the template for PCR amplification. The PCR product for *dndB* was 365 bp, and *rrnA* encoding 16s rRNA was used as the internal control. Forward and reverse primers are listed in Table S2. PCR products were examined by electrophoresis on a 1.2% agarose gel and visualized by staining with ethidium bromide.

For quantitative real-time PCR, reactions were performed on an ABI7500 Fast Real Time System (Applied Biosystems). A standard reaction mixture (25 μl) contained 1 or 150 ng cDNA (1 ng for reference gene, 150 ng for target gene), 12.5 μl SYBR Green qPCR Master Mix (Thermo Fisher Scientific Inc.), and 50 nM of the forward and reverse primers. The PCR product for *dndB* was 161 bp, and transcript amounts were normalized to the amount of 16s rRNA. The conditions for PCR amplification were 95°C for 10 min, followed by 40 cycles of 95°C for 15 s, 60°C for 1 min. A dissociation curve ranging from 55°C to 90°C in 0.5°C increments, with a dwell time of 30 s, was performed to assess the specificity of the reactions.

### Determination of the transcriptional start site of the *dnd* gene cluster

The 5′ RACE System Kit (ThermoFisher) was used to determine the transcriptional start sites of the *dnd*A and *dnd*B genes, according to protocols provided by the manufacturer. RNA was isolated from *S. lividans* 1326 grown in TSBY medium. First-strand cDNA was synthesized using SuperScript reverse transcriptase and a gene-specific primer, GSP1 (A-GSP1 for *dnd*A, B-GSP1 for *dnd*B). RNA was removed by treatment with RNase Mix, and the cDNA was purified on a S.N.A.P Column (ThermoFisher). A poly(dC) tail was added to the 3′-end of the cDNA using terminal deoxynucleotidyl transferase and dCTP. The first PCR amplification was performed using *rTaq* polymerase (TAKARA BIO INC.), a nested, gene-specific primer, GSP2 (A-GSP2 for *dnd*A, B-GSP2 for *dnd*B), which anneals to a site within the cDNA, and AAP (Abridged Anchor Primer). The second PCR amplification used AUAP (Abridged Universal Amplification Primer) with another nested, gene-specific primer, GSP3 (A-GSP3 for *dnd*A, B-GSP3 for *dnd*B), the sequence of which is located upstream of GSP2 and within the cDNA product. The product of the second PCR was ligated into pMD18-T vector for sequencing.

### RNA-seq and data analysis

Spores of the wild type *S. lividans* 1326 and the HXY6 were grown in TSBY media to an OD_450_ reading of 0.3. The cultures were then collected by centrifugation and total RNA was isolated using RNAprotect Bacteria Reagent and RNeasy Mini kits (Qiagen), followed by further purification using a MICROBExpress kit (Ambion) to enrich the mRNA. The resulting mRNA was fragmented using an RNA fragmentation kit (Ambion), producing fragments of sizes ranging from 200 bp to 250 bp. Double-stranded cDNA was generated using the SuperScript Double-Stranded cDNA Synthesis Kit (Invitrogen) and purified using a QiaQuick PCR extraction kit. An RNA-seq library was constructed using a Paired End Sample Prep kit (Illumina), and the sequencing was performed using the HiSeq 2000 sequencer (Illumina). Raw reads were filtered to remove dirty reads as recommended by Illumina, and then the resulting clean reads were analyzed with TopHat (version 2.0.5) software (Langmead et al., 2009), using the annotated *S. lividans* 1326 genome as reference. We employed R packages edgeR (version3.3; Robinson et al., 2010) for differential gene expression analysis in this study, using multiple hypothesis test for the *p*-value correction (*q*-value), and taking in to account the false discovery rate (FDR). Fold-change was calculated according to the FPKM (Fragments Per Kilobase of exon model per Million mapped reads) value (screening criteria *q*-value 2 ≤ 0.05, and Fold-change ≥ 2). Gene Ontology (GO) enrichment analysis of differentially expressed genes was analyzed using the GOseq R package, with gene length bias corrected (Young et al., 2010). GO terms with *q*-value < 0.05 were considered significantly enriched by differentially expressed genes.

## Results

### Anti-oxidation advantage of DNA phosphorothioate modification in *S. lividans*

**Figure 1:**
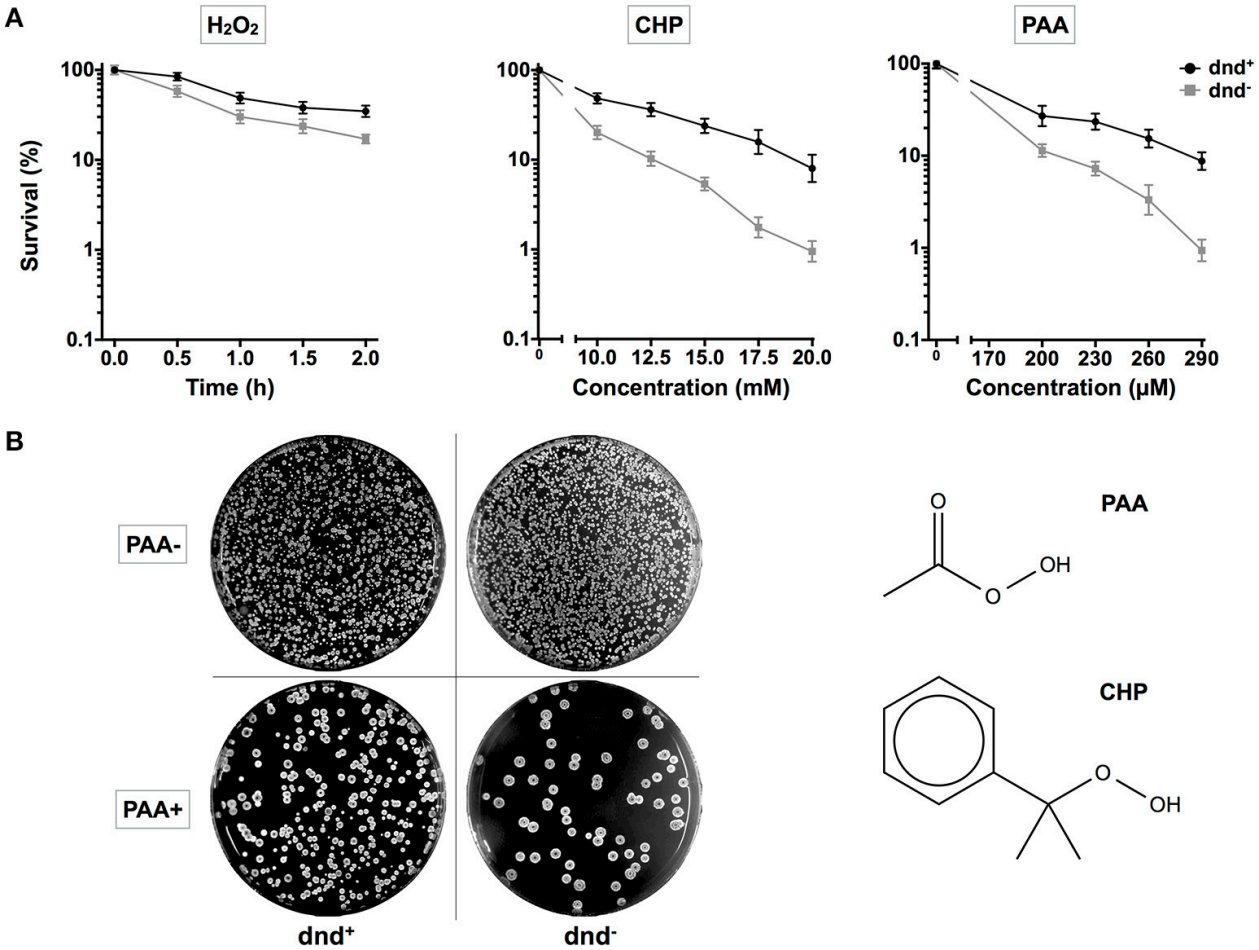
**Survival of ***S. lividans*** under oxidative stress**. **(A)** Survival rate of the *S. lividans* 1326 wild type strain and the phosphorothioate modification negative mutant (*dnd*^−^) following treatment with 20 mM H_2_O_2_ (0.5, 1, 1.5, or 2 h), CHP (10–20 mM), and PAA (200–290 μM). **(B)** Plates showing colonies before and after the treatment with 290 μM PAA for 30 min. The chemical structures of PAA and CHP are shown. Three replicates were performed.

To investigate the potential consequences for host cells, germinated spores of *S. lividans* 1326 wild type (WT) strain and its mutant daughter strain HXY6, from which the *dnd* gene cluster was removed, were treated with H_2_O_2_, PAA, or CHP. PAA and CHP were tested as representatives of organic hydroperoxides. After treatment, the spores were spread onto agar plates, and colonies were counted to calculate the survival rates (Figure 1). WT had a two-fold higher survival rate than HXY6 following H_2_O_2_ treatment, and a 10-fold higher survival rate than HXY6 following treatment with 20 mM CHP or 290 μM PAA (using mycelia, similar result was obtained for PAA treatment, Figure S1). These observations suggest that phosphorothioation of the DNA confers an anti-oxidation survival advantage to the host.

### Global transcriptional response of *S. lividans* following loss of DNA phosphorothioation

**Figure 2:**
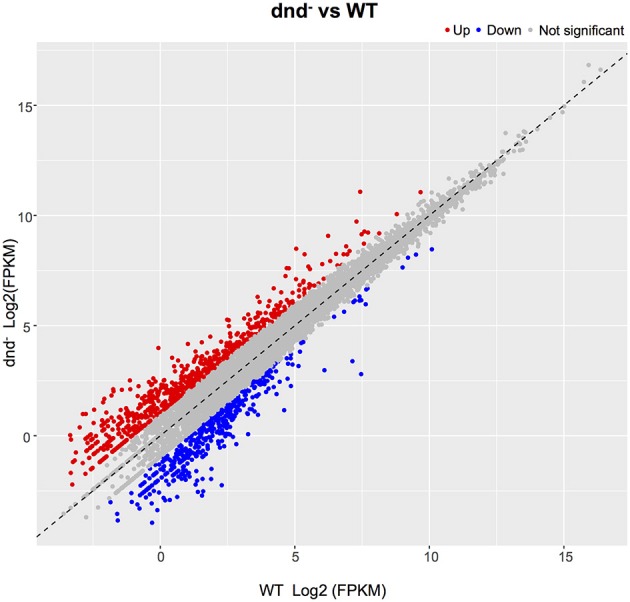
**Gene expression differences between the WT and ***dnd***^**−**^ strains**. The numbers of differentially expressed genes between WT and the *dnd*^−^ mutant are indicated. Genes were determined using the publicly available *S. lividans* 1326 genome. When compared to the control, genes with a log2 fold change >1.0, and FDR-adjusted *p*-values ≤ 0.05, were considered to be differentially expressed.

**Figure 3:**
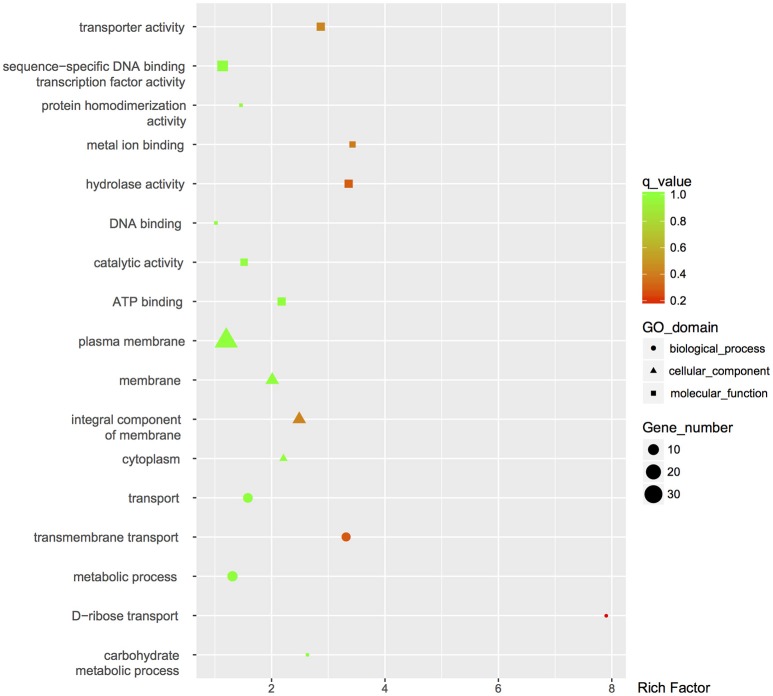
**GO enrichment analysis of differentially expressed genes**. GO terms with *q*-value < 0.05 were considered significantly enriched by differentially expressed genes.

The global change in gene expression analysis in response to phosphorothioation modification was conducted to investigate whether DNA phosphorothioation up-regulates genes that express peroxide scavengers and that contribute to the anti-oxidative properties associated with the modification. The mRNA levels of each gene were compared between WT and the *dnd*^−^ mutant HXY6. A total of 206 genes had significantly different expression, as measured by a 2-fold (log2- ratio. 1, *p*-value = 0.05) or greater difference between the two strains, with 146 genes up-regulated (Table S3) and 60 genes down-regulated (Table S4) in the mutant (Figure 2). Gene Ontology enrichment analysis shows that the differentially expressed genes scattered to seventeen pathways (or molecular functions), and none of them has *p*-value lower than 0.05 and at the same time contains more than 5 genes (Figure 3). Although a bunch of transcriptional regulators were seen in the differential expressed genes (Tables S3, S4), homology analysis of these genes indicated that these genes are scattered to multiple different pathways. Gene Ontology enrichment analysis also indicated that none of these regulator genes has *p*-value lower than 0.05 and concentrate in pathway containing more than 5 genes. These observations suggested that the global RNA transcriptions of the two strains are essentially similar. Particularly, differences of anti-oxidative genes (including catalase, alkyl hydroperoxide reductase CD, and organic hydroperoxide resistance genes) between the two strains were not seen. The resistance to hydrogen peroxide conferred by DNA phosphorothioation might not involve up-regulation of these anti-oxidation genes in advance.

### Transcriptional response of catalase and hydroperoxide resistance genes following peroxide treatments

**Figure 4:**
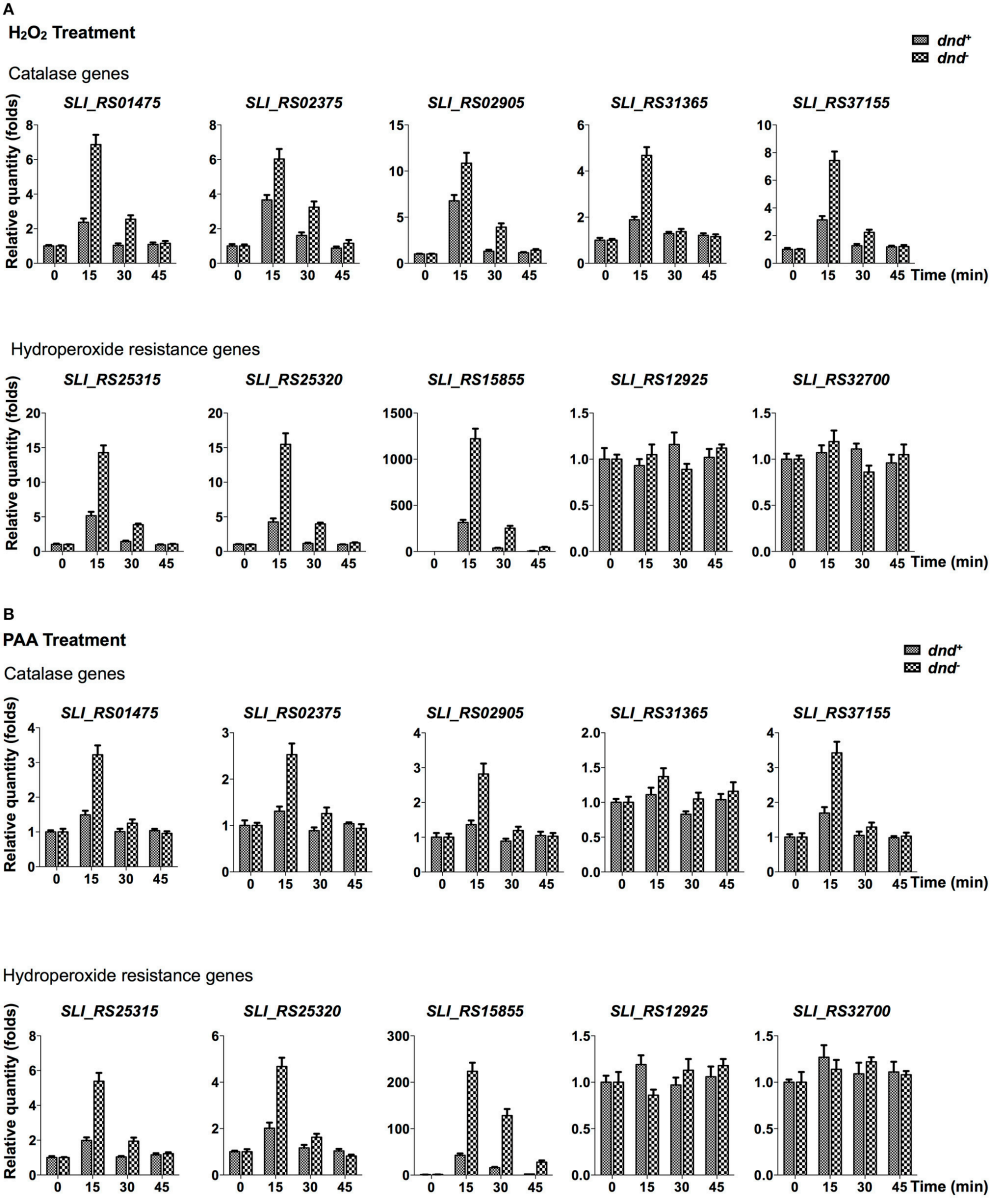
**Quantitative RT-PCR analysis of peroxide scavenging gene response to peroxides**. *S. lividans* cells from *dnd*^+^ or *dnd*^−^ strains were grown to an OD_450_ of 0.3 before addition of 20 mM H_2_O_2_ **(A)**, or 200 μM PAA **(B)**. At the indicated time intervals, cells were collected and the transcription of catalase, alkylhydroperoxidase reductase, and hydroperoxide resistance genes were measured using quantitative RT-PCR (the reference value for fold change is time zero). The *rrn*A gene was set as the internal standard. Three replicates were performed.

Quantitative RT-PCR analysis was conducted to characterize the transcriptional response of peroxide scavenging genes following the loss of DNA phosphorothioation. There are five catalase (*SLI_RS01475, SLI_RS02375, SLI_RS02905, SLI_RS31365*, and *SLI_RS37155*), two alkylhydroperoxidase reductases (*SLI_RS25315* and *SLI_RS25320*), and three organic hydroperoxide resistance genes (*SLI_RS15855, SLI_RS12925*, and *SLI_RS32700*) in the genome. Transcriptional changes of these genes were traced in 45 min after peroxide treatments (Figure 4, and Figure S2 for the removal of hydrogen peroxide).

Figure 4A shows that the five catalase, and two alkylhydroperoxidase reductase genes were activated by 20 mM hydrogen peroxide, reaching peaks in 15 min after the treatment. Two organic hydroperoxide resistance genes (*SLI_RS12925* and *SLI_RS32700*) did not response to the hydrogen peroxide treatment. Notably, *Sli_RS15855*, the homolog of ohr in *S. coelicolor*, was up-regulated hundreds of folds in 15 min. The eight responsive genes were more activated in the *dnd*^−^ mutant than the wild type strain. Similar responses were also observed for the PAA treatment (Figure 4B, and Figure S2 for the removal of PAA).

### DNA phosphorothioate modification as a peroxide-scavenging system in *S. lividans*

The observation that catalase and organic hydroperoxide resistance genes (thiol-dependent oxidase; Cussiol et al., 2003, 2010), were more induced in the *dnd*^−^ strain by H_2_O_2_and PAA treatments, reaching levels higher than the wild type, suggested that the DNA phosphorothioation system compensated for the increased catalase and peroxidase activity present in the mutant.

**Figure 5:**
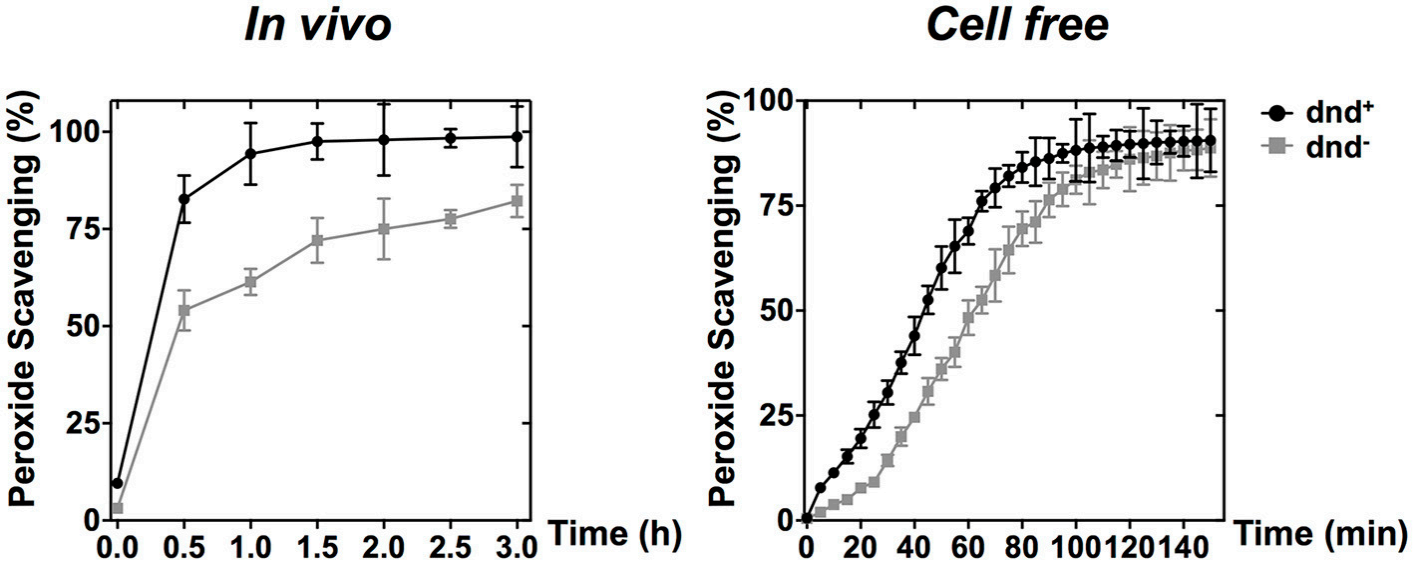
**PAA scavenging activity of phosphorothioate modification**. The curves show the removal (scavenging) of PAA by wild type and the *dnd*^−^ mutant cells (*in vivo*), or the corresponding whole cell lysates (*in vitro*). Concentrations of 500 μM and 5 mM were used for the *in vivo* and *in vitro* scavenging reactions, respectively. Three replicates were performed.

PAA, which is an organic hydroperoxide, was used to test whether the WT strain can scavenge the peroxide directly. Results showed that PAA was scavenged more rapidly by WT than by the *dnd*^−^ mutant, both *in vivo* (500 μM) and *in vitro* (5 mM) immediately after peroxide exposure (Figure 5), supporting the suggestion that the modification system constitutes a new type of organic hydroperoxide resistance system in *S. lividans*.

### Determination of the *dnd* promoter

Determination of the transcriptional response of the *dnd* promoter to oxidants might provide additional evidence for its physiological function as an antioxidation system.

**Figure 6:**
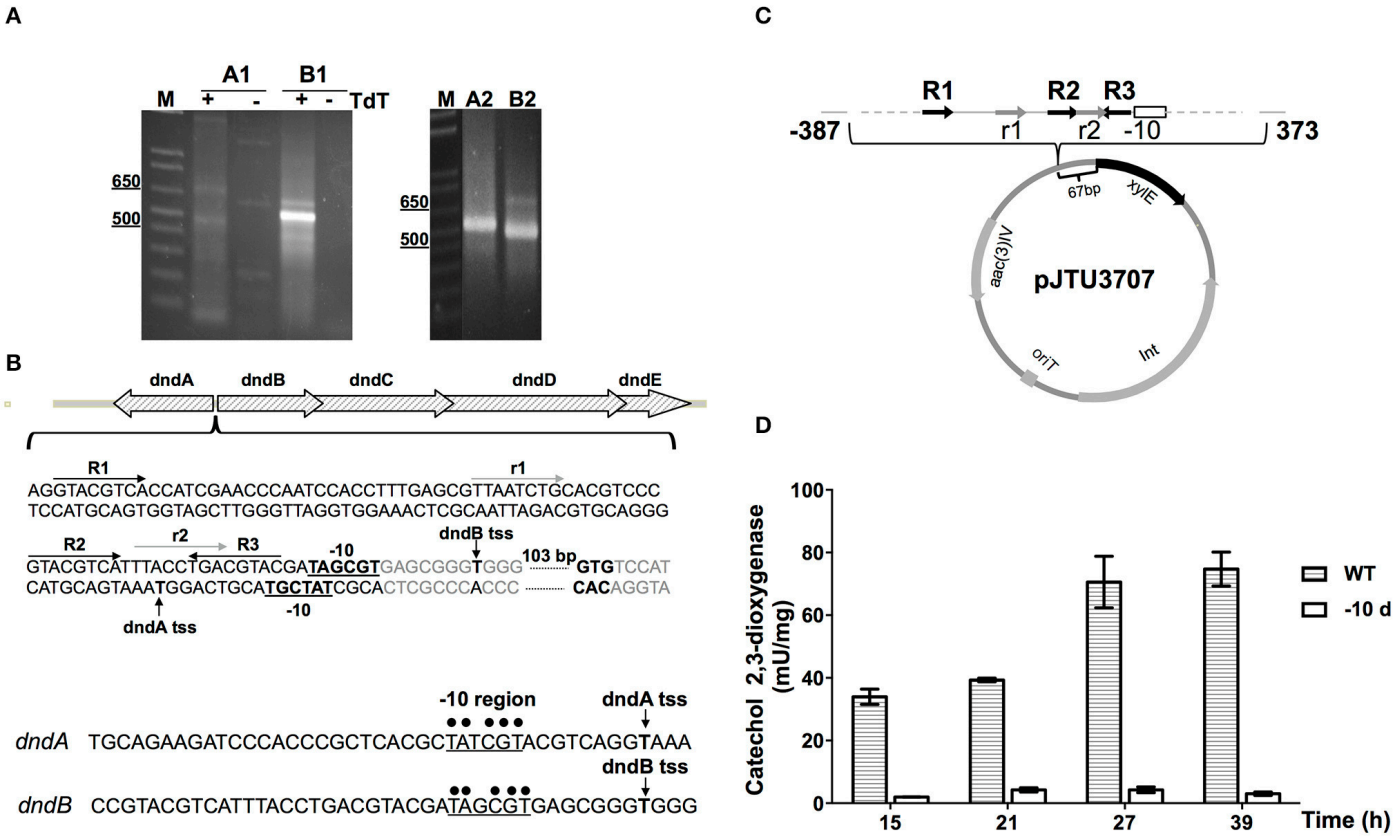
**Identification of the ***dnd*** promoter. (A)** The transcriptional start sites (tss) of *dndA* and *dndB*, which are divergently transcribed genes, were determined using 5′ RACE. The promoters were analyzed using two rounds of PCR, and A1 and A2, and B1 and B2, indicate the products from the first and second round of PCR for the *dnd*A and *dnd*B genes, respectively. **(B)** Schematic diagram of the *dnd* promoter region. The repeats R1, R2, and R3 are marked by dark arrows, and the direct repeats r1 and r2 are marked by light gray arrows. The -10 regions are in bold and underlined, and the tss are indicated by vertical arrows. Promoters of *dnd*A and *dnd*B were aligned with the consensus sequence for the -10 region, which is marked by dots. **(C)** Diagram of pJTU3707 showing the structure of the *xylE* reporter plasmid. **(D)** The catechol 2,3-dioxygenase activity assay was used to monitor the effect of the deletion of the -10 region on activity from the *dndB* promoter.

5′ RACE was conducted first to determine the transcriptional start site of the *dnd* gene cluster. Gene specific primers were designed to amplify fragments of cDNA of *dndA* and *dndB*. After the addition of poly(dC) tails, PCR was performed, and the second round of nested PCR generated clear amplification products (Figure 6A). The fragments were then cloned and sequenced, obtaining the transcriptional start sites of *dnd*A and *dnd*B. The -10 regions of *dnd*A and *dnd*B promoters are quite similar, containing the consensus sequence TA(t/g)CGT. Two kinds of repeat sequences, the R repeat (R1-R2-R3) and the r repeat (r1-r2) were also found in the -35 region (Figure 6B).

The location of the *dnd* promoter was further characterized using the *xyl*E reporter gene. A DNA fragment containing the region from −387 to 373 bp with respect to the *dnd* transcriptional start site was inserted in front of the *xyl*E gene (Figure 6C), and to measure *xylE* expression levels, total protein was extracted and catechol 2,3-dioxygenase activity was assayed. Two strains were compared, the WT and a mutant from which the -10 region of the *dndB* promoter was removed from upstream of *xylE*. As shown in Figure 6D, with the deletion of the -10 region from the *dnd*B promoter, the catechol 2,3-dioxygenase activity dropped sharply compared to the wild type control.

### Up-regulation of the *dnd* gene transcription by diamide

**Figure 7:**
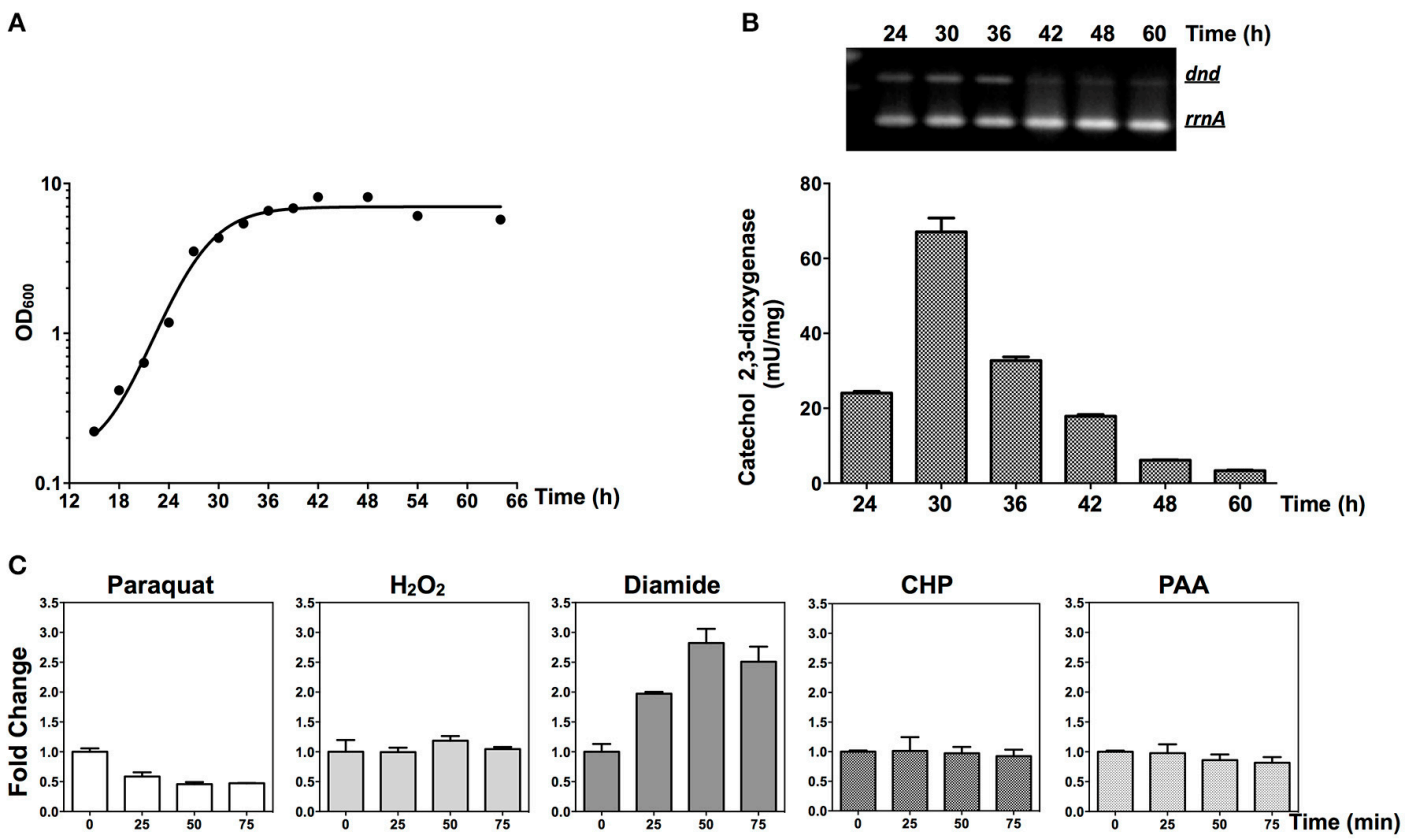
**Transcriptional responses of ***dnd*** to oxidants. (A)** Growth curve of the wild type strain. **(B)** The time course of transcription of the *dnd* gene cluster analyzed using semi-quantitative RT-PCR and the catechol dioxygenase activity assay. For the semi-quantitative RT-PCR, the wild type strain was used and for the catechol dioxygenase activity assay, the wild type strain containing pJTU3707 was used. **(C)** Quantitative RT-PCR analysis of *dndB* transcription following treatment with 1 mM paraquat, 20 mM H_2_O_2_, 3 mM diamide, 3 mM CHP, or 200 μM PAA (the reference value for fold change is time zero). The *rrnA* gene was set as the internal control for evaluating transcriptional activity from the *dndB* promoter.

RNA was extracted and RT-PCR was performed to monitor the transcription of the *dnd* gene cluster. As shown in Figures 7A,B, the *dnd* gene cluster is transcribed early during growth, corresponding to log phase growth of the strain. The early expression of the *dnd* gene cluster was further confirmed using the *xyl*E reporter construct (Figures 6B,C,7B).

At the mid-log phase of growth, the wild type strain was treated using 20 mM H_2_O_2_, 1 mM paraquat, 3 mM CHP, 3 mM PAA, and 3 mM diamide, the concentrations for which were chosen based on published studies (Lee et al., 1993; Kallifidas et al., 2010). These are model compounds that can induce the expression of superoxide dismutase, catalase, and organic hydroperoxide resistance genes. Diamide is special for its capability that can induce the expression of both superoxide dismutase, and catalase genes via thiol-based switches (Privalle et al., 1993; Zheng et al., 1998; Antelmann and Helmann, 2011). RNA was then isolated and quantitative RT-PCR was conducted to determine whether the *dnd* transcription is subjected to oxidant regulation. Transcription from the *dnd* gene cluster decreased slightly after 1 mM paraquat treatment (Figure 7C), but with H_2_O_2_, CHP, and PAA treatment, there was no obvious change in transcription within the same time period of 75 min (lower concentrations from 100 μM to 1 mM were also tried, with similar results observed). However, with diamide treatment, transcription increased about 2.5 fold in 50 min.

**Figure 8:**
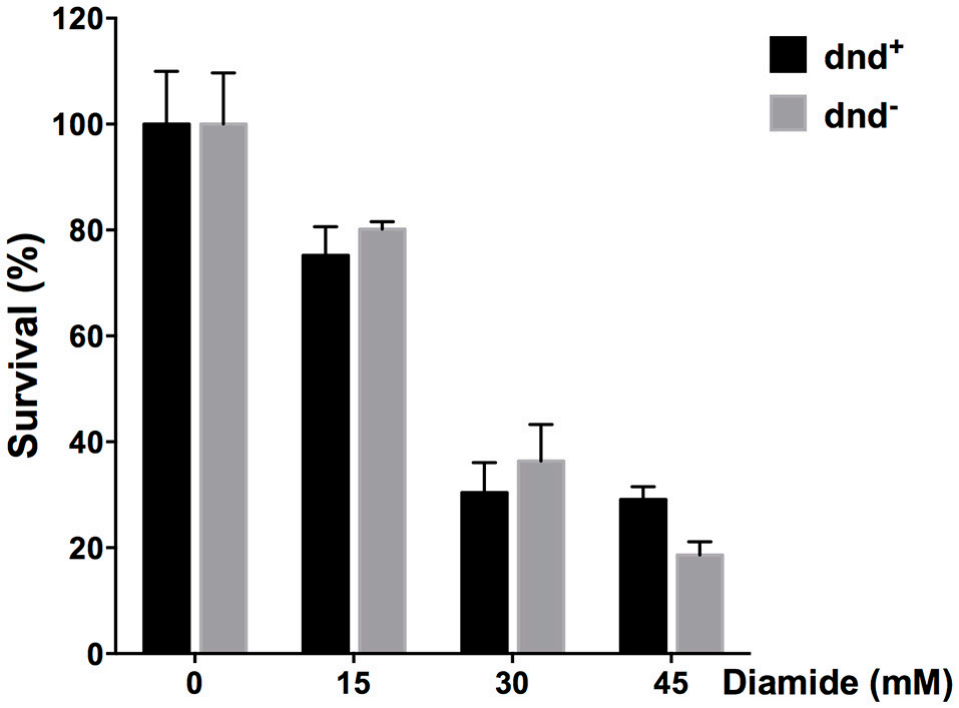
**Survival of ***S. lividans*** under diamide stress**. Survival rate of the *S. lividans* 1326 wild type strain and the *dnd*^−^ mutant following treatment up to 45 mM diamide for an hour. Three replicates were performed.

Since diamide induced the expression of *dnd* genes, we tested the survival of the strains under diamide stress. Figure 8 shows that there arenÕt significant differences between WT and the *dnd*^−^ mutant.

**Figure 9:**
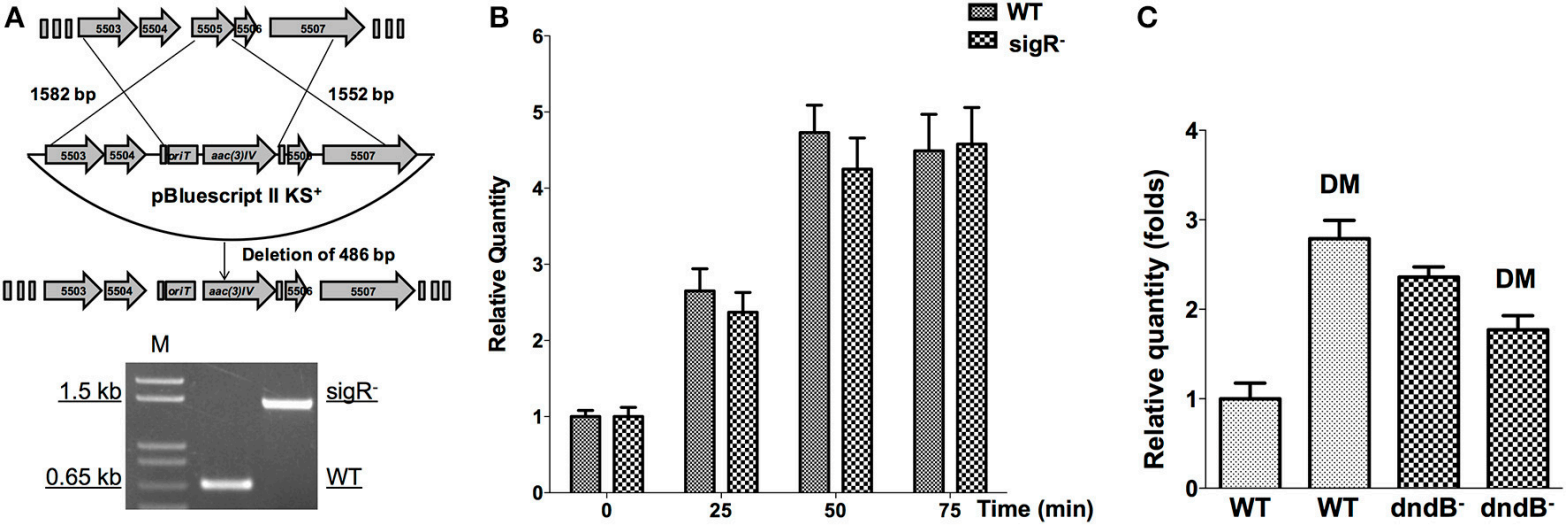
**Potential regulators of ***dnd*** gene cluster in response to diamide**. **(A)** The sigR gene was disrupted using an apramycin resistance gene cassette, confirmed by PCR. Transcriptional response of *dnd* gene cluster to diamide (3 mM) was compared between the wild type and sigR^−^ mutant **(B)**. For comparison between the wild type and *dnd*B^−^ mutant, samples were collected at 45 min **(C)**.

In *S. coelicolor*, a sigmaR-RsrA regulation system composing of a sigma factor and a redox sensor anti-sigma factor (Paget, 1998; Kang et al., 1999), responds to diamide treatment. We disrupted *sig*R gene, and then tested whether *dnd* transcription is subjected to SigR regulation. Figures 9A,B shows that there is no obvious *dnd* gene expression difference between WT and the *sig*R^−^ mutant after diamide treatment. Therefore, the response to diamide is not mediated by SigR.

DndB is the repressor of *dnd* gene cluster. The possibility of DndBÕs response to diamide was explored. Figure 9C shows that in *dnd*B in-frame deletion mutant, the response to diamide stimulation is lost. DndB might therefore participate in the diamide induction process.

## Discussion

*Streptomyces* species are highly adapted to living in soil, an environment with an extremely diverse microbial composition, and where competition between microbes for organic components is expected. Since reactive oxygen species (ROS), such as superoxide (O^2−^), H_2_O_2_, and the hydroxyl radical (OH•) can damage lipids, proteins, and DNA, and cause various cellular lesions (Imlay, 2013), their destructive properties have been harnessed by organisms as a weapon against other species (Geiszt and Leto, 2004; Lambeth, 2004). For example, NADPH oxidase, which generates ROS, is highly conserved across virtually all multicellular life (Lambeth and Neish, 2014). Additionally, lactic acid bacteria suppress the growth of competing microbes by using pyruvate and lactate oxidases to excrete large doses of H_2_O_2_ (Imlay, 2008; Martín and Suárez, 2010). Recently, it was shown that actinorhodin, a natural product synthesized by *S. coelicolor*, can catalyze the production of toxic levels of H_2_O_2_, and inhibit bacterial growth via the generated H_2_O_2_ (Nishiyama et al., 2014). Therefore, it seems that the soil environment is a battlefield where ROS is a formidable weapon.

Interestingly, anti-oxidation systems are also apparently abundant in *Streptomyces*. There are multiple catalases, thiol peroxidases, alkylhydroperoxide reductases (Ahp), and organic hydroperoxide resistance protein homologs in *S. lividans*, which are expected to have antioxidation activities. It is suspected that DNA phosphorothioate modification also functions as an anti-oxidation system in *S. lividans*, and, in this work, we provided several lines of evidence supporting this role. First, the absence of DNA phosphorothioation increases sensitivity to the peroxides H_2_O_2_, PAA, and CHP (Figure 1). Secondly, there is a distinctive difference in the PAA-scavenging activity between the wild type and *dnd*^−^ mutant strains, and thirdly, the *dnd* promoter is subject to oxidative (diamide) regulation.

Hydrogen peroxide and organic hydroperoxides are different types of oxidants. Physiologically, organic hydroperoxides, such as lipid hydroperoxide, are prominent non-radical products generated in the process of unsaturated fatty acid-initiated lipid peroxidation under oxidative stress (Girotti, 1998). The hydroperoxides are scavenged by organic hydroperoxide resistance proteins in bacteria using thiol or lipoyl group as the reductant (Cussiol et al., 2003, 2010). Although bacteria synthesize only saturated or monounsaturated fatty acids, many bacteria will take up exogenous polyunsaturated fatty acids if they are presented in the culture medium (Watanabe et al., 1994). The existence of organic hydroperoxide resistance genes suggests that the stress exists in *S. lividans*.

The special anti-oxidative advantage conferred by phosphorothioate modification may be the organic hydroperoxide resistance. The viewpoint is supported by the observation of the 10-fold difference in survival between the wild type strain that has DNA phosphorothioate modification and the *dnd*^−^ mutant, upon PAA or CHP treatment (Figure 1). Another clue is that *SLI_RS15855* was induced highly in the *dnd*^−^ mutant following 20 mM H_2_O_2_ or PAA treatment (Figure 4). The higher induction of *ohr*A in the *dnd*^−^ mutant suggested that more organic hydroperoxides accumulated in the mutant than the wild type.

Two significant questions remain to be addressed: firstly, how the scarcely phosphorothioated sites counteract the peroxides by orders of folds; secondly, why the modified DNA bonds were not cleaved by peroxides, especially by PAA *in vivo*. The turning-over model (recycling of the phosphorothioate sulfur), as suggested previously (Xie et al., 2012), could explain the first question very well, but not the second one since the result of reaction with PAA of the bond *in vitro*, is DNA degradation, which should be lethal to the bacteria. An alternative suggestion is that the peroxide-scavenging behavior of the phosphorothioate might be a well-controlled catalytic process, implying the involvement of enzymes. The potential enzymes are Dnd proteins: the protein themselves or the protein-phosphorothioate DNA complex might scavenge the peroxides.

The *dndBCDE* gene cluster constitutes a polycistronic transcriptional unit (Xu et al., 2009). The cluster is actively transcribed in the early stage of exponential growth, possibly because DNA is replicated in this stage. Interestingly, the *dnd* gene cluster was induced by diamide, which can cause disulfide stress in cells. Synthesized phosphorothioate can form disulfide bond (Wu and Orgel, 1991). The survival rate of the wild type and the *dnd*^−^ mutant strain treated by diamide exhibited no difference (Figure 8), suggesting that DNA phosphorothioate modification might not scavenge disulfide bonds *in vivo*. Induction of the *dnd* gene cluster by diamide suggests that the underlying activation mechanism of the gene cluster might be the formation of disulfide bonds, which is reminiscent of OxyR (Zheng et al., 1998), a regulator of oxidative genes including catalases (Imlay, 2008). Similarly, in *S. coelicolor*, transcriptional regulation of *cat*A by CatR, is also Redox-dependent, with the involvement of thiol oxidation in redox modulation of CatR (Hahn et al., 2000).

With *S. coelicolor*, the cells responded to disulfide stress through a sigmaR-RsrA regulation system, composed of a sigma factor and a redox sensor anti-sigma factor (Paget, 1998; Kang et al., 1999). SigmaR regulates promoters containing the conserved sequences GGAAT and GTTG at the −35 and −10 regions, respectively, (Paget, 1998; Kang et al., 1999), but these two sequences were not observed in the *dnd* promoter of *S. lividans*. Indeed, significant differences of transcriptional response to diamide were not observed between a sigR mutant and wild type (Figures 9A,B). A potential disulfide-responsive regulator of *dnd* genes might be DndB, which is a negative regulator of DNA phosphorothioation (Cheng et al., 2015; He et al., 2015). Quantitative RT-PCR analysis shows that in a *dnd*B deletion mutant, the transcriptional activation by diamide of *dnd* gene cluster was lost (Figure 9C). The possible mechanism might be that Diamide induced disulfide inactivate DndB and allow more transcription of the *dnd* gene cluster.

Overall, the observations suggested that DNA phosphorothioate modification in *S. lividans* is a peroxide resistance system.

## Author contributions

DD, AD, KX, and TP did the experiments. DD, AD, TP, XZ, ZD, JL, XH, and ZW analyzed the data. DD, XZ, ZD, JL, XH, and ZW wrote the main manuscript text. DD, JL, and ZW prepared figures. All authors reviewed the manuscript.

### Conflict of interest statement

The authors declare that the research was conducted in the absence of any commercial or financial relationships that could be construed as a potential conflict of interest.

## Supplementary material

Figure S1 **Survival rate of PAA treatment**. Mycelia of *S. lividans* WT and the *dnd*^−^ mutant were treated using increasing concentration of PAA. The cells were treated for 1 h. Then the colonies were counted to calculate the survival rates. The experiments were repeated three times.

Figure S2 **Removal of H_**2**_O_**2**_ and PAA**. Mycelia of *S. lividans* WT and the dnd- mutant were treated using 20 mM H_2_O_2_ or 200 μM concentration of PAA. At indicated intervals, the remains of the peroxides were monitored.

Table S1 **Strains and plasmids used in the study**.

Table S2 **Primers used in this study**.

Table S3 **Up-regulated genes in the mutant**.

Table S4 **Down-regulated genes in the mutant**.

## Abbreviations

PAA: peracetic acid
CHP: cumene hydroperoxide
OD: optical density.
