# Supplementary figures and images for: DNA Phosphorothioate Modification Plays a Role in Peroxides Resistance in Streptomyces lividans
Source: Front Microbiol. 2016 Aug 31;7:1380. doi: 10.3389/fmicb.2016.01380 (PMC5005934; doi:10.3389/fmicb.2016.01380)

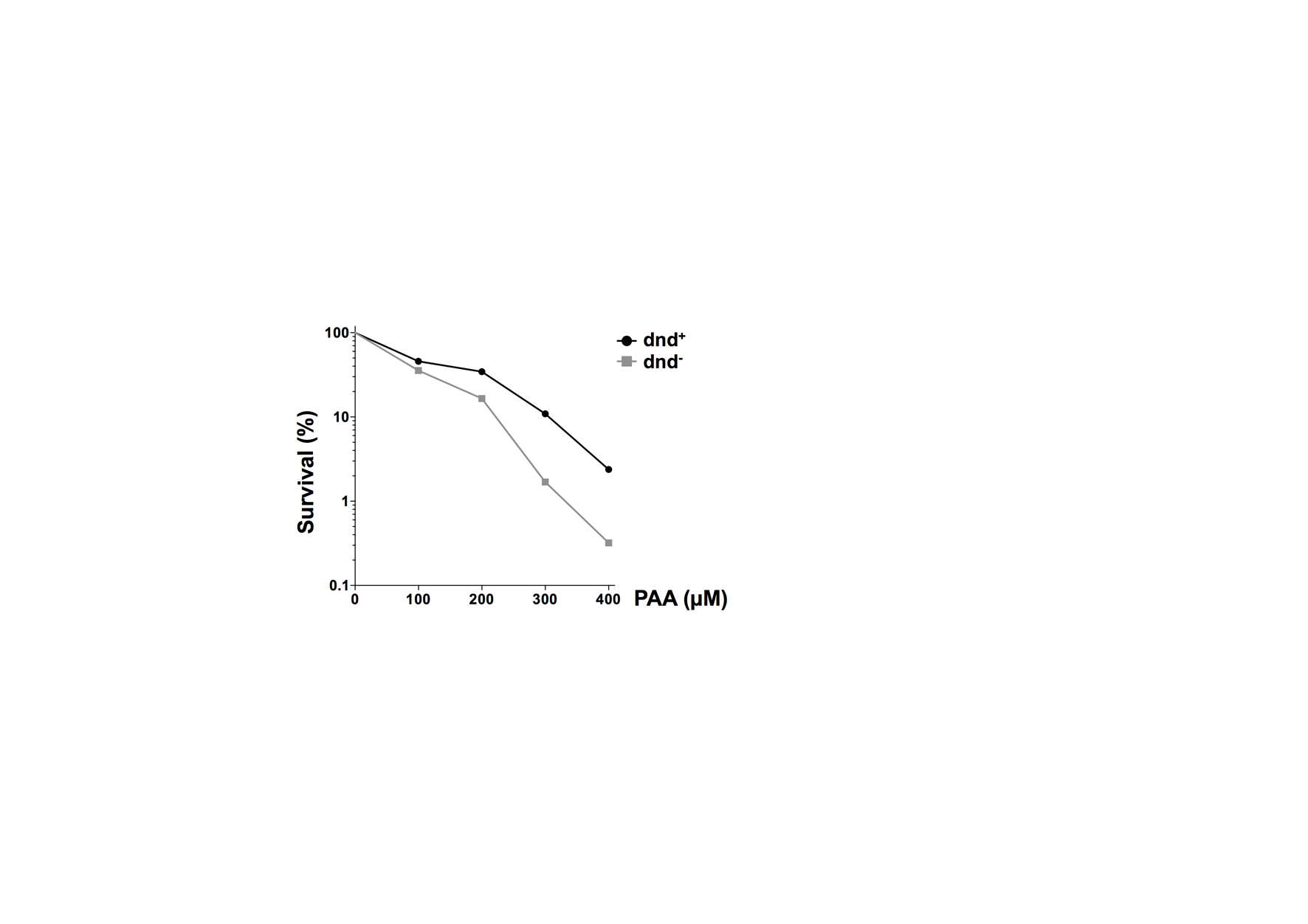

Supplement: Figure S1 — Survival rate of PAA treatment. Mycelia of S. lividans WT and the dnd− mutant were treated using increasing concentration of PAA. The cells were treated for 1 h. Then the colonies were counted to calculate the survival rates. The experiments were repeated three times. [file Image1.TIFF]

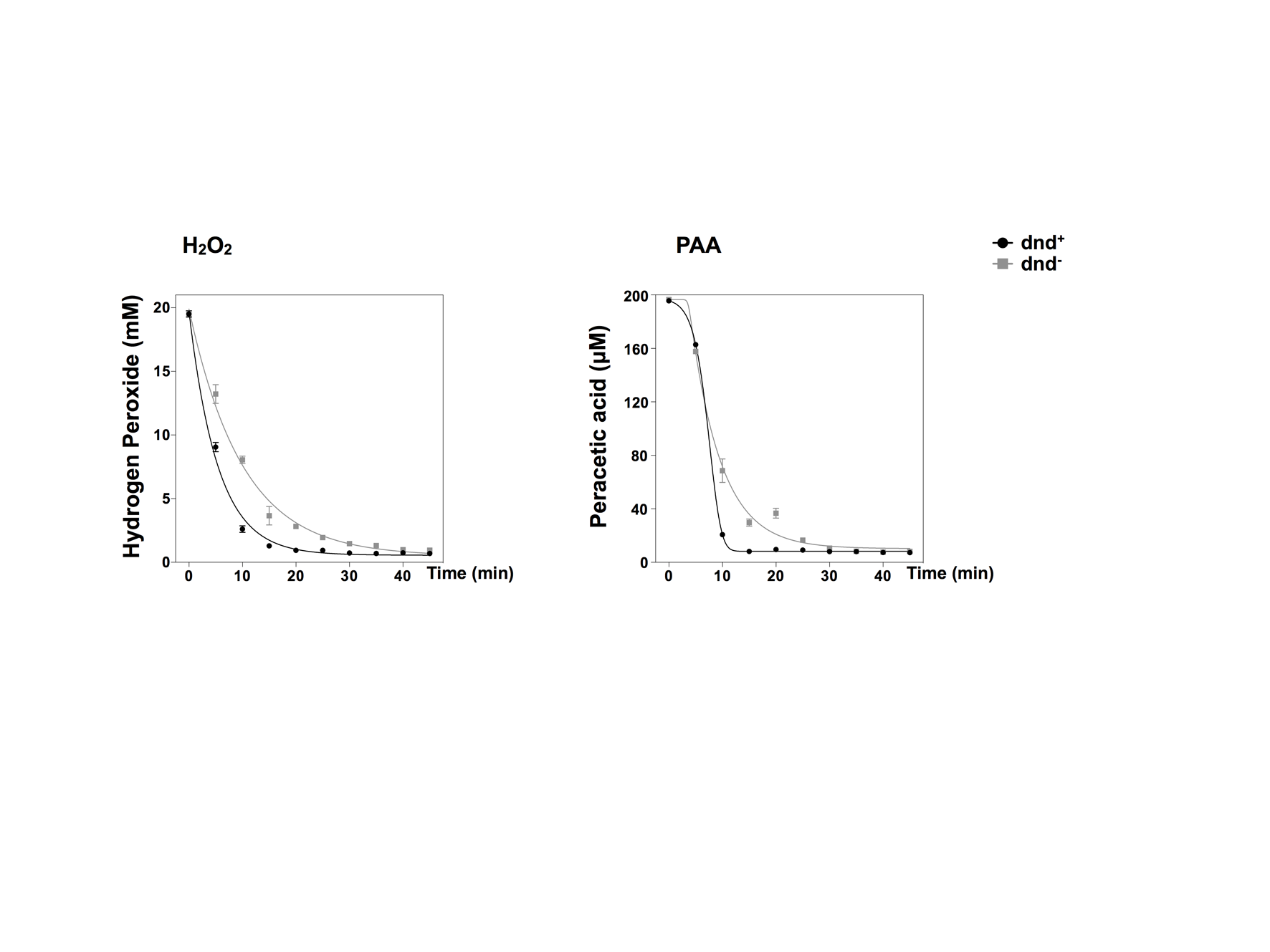

Supplement: Figure S2 — Removal of H2O2 and PAA. Mycelia of S. lividans WT and the dnd- mutant were treated using 20 mM H2O2 or 200 μM concentration of PAA. At indicated intervals, the remains of the peroxides were monitored. [file Image2.TIFF]
