# Supplementary material for: DNA Phosphorothioate Modification Plays a Role in Peroxides Resistance in Streptomyces lividans
Source: Front Microbiol. 2016 Aug 31;7:1380. doi: 10.3389/fmicb.2016.01380 (PMC5005934; doi:10.3389/fmicb.2016.01380)
Supplement: Table S1 — Strains and plasmids used in the study. [file Table1.DOC]

# Supplementary Materials for

# Peroxide Resistance of DNA Phosphorothioate Modification in *Streptomyces lividans*

Daofeng Dai, Aiqin Du, Kangli Xiong, Tianning Pu, Xiufen Zhou, Zixin Deng, Jingdan Liang^#^, Xinyi He^#^, and Zhijun Wang^#^

State Key Laboratory of Microbial Metabolism and School of Life Science & Biotechnology,

Shanghai Jiao Tong University, Shanghai 200030, China

For correspondence:

Email: wangzhijun@sjtu.edu.cn

Table S1

| Strains and plasmids | Description | Source/reference |
| --- | --- | --- |
| **Strains** |  |  |
| *S. lividans* 1326 | Wild type, dnd^+^, SLP2+, SLP3+ | [1] |
| HXY6 | dnd^-^, the whole *dnd* gene cluster deletion mutant | [2] |
| HXY2 | dndB^-^, dndB in-frame deletion mutant | [2] |
| DDF1 | sigR^-^, sigR disruption mutant,  *apr^r^* | This study |
| *E. coli* DH5α | F^-^, *recA*, *lacZ*, ∆M15 | [3] |
| E. coli ET12567  /pUZ8002 | pUZ8002, *recF*, *dam*, *dcm*, *hsds*, *cml^r^*, *km^r^* | [4] |
| **Plasmids** |  |  |
| pSET152 | *aac(3)*, *lacZ*, *rep^pUC^*, *att^ΦC31^*, *oriT* | [5] |
| pIJ4083 | *xylE*, *tsr*, *ori^pIJ101^* | [6] |
| pIJ702 | pIJ101 derivative, *tsr mel* | [7] |
| pJTU3700 | Insertion of *xylE* from pIJ4083 into pSET152 | This work |
| pJTU3707 | Insertion of P*dnd*B into pJTU3700 | This work |
| pJTU3707-10 | Deletion of -10 region of P*dnd*B in pJTU3707 | This work |
| pMD18-T | Vector for DNA sequencing, *bla* | Takara |
|  |  |  |

Table S1. Strains and plasmids used in the study

References

1. Zhou, X. *et al. Streptomyces coelicolor* A3(2) lacks a genomic island present in the chromosome of *Streptomyces lividans* 66. *Appl. Environ. Microbiol.* 70, 7110-8 (2004).

2. Liang, J. *et al.* DNA modification by sulfur: analysis of the sequence recognition specificity surrounding the modification sites. *Nucleic Acids Res.* 35, 2944–2954 (2007)

3. Hanahan, D. *Echerichia coli* with plasmids. *J. Mol. Biol.* 166, 557-580 (1983).

4. Flett, F. *et al.*High efficiency intergeneric conjugal transfer of plasmid DNA from *Echerichia coli* to methyl DNA-restricting streptomycetes. *FEMS Microbiol. Lett.* 155, 223-9 (1997).

5. Bierman, M. *et al.*Plasmid cloning vectors for the conjugal transfer of DNA from *Echerichia coli* to *Streptomyces spp. Gene* 166, 43-9 (1992).

6. Timothy, C. *et al. Streptomyces* promoter-probe plasmids that utilise the *xylE*

gene of *Pseudomonas putida*. *Nucleic Acids Res.* 18, 1077 (1989).

7. Zhu, D. *et al*. Expression of the *mel*C operon in several *Streptomyces* strains is

positively regulated by *adp*A, an *ara*C family transcriptional regulator involved in morphological development in *Streptomyces coelicolor.* J. Bacteriol. 3180–3187 (2005)
