# Supplementary material for: DNA Phosphorothioate Modification Plays a Role in Peroxides Resistance in Streptomyces lividans
Source: Front Microbiol. 2016 Aug 31;7:1380. doi: 10.3389/fmicb.2016.01380 (PMC5005934; doi:10.3389/fmicb.2016.01380)
Supplement: Table S2 — Primers used in this study. [file Table2.DOCX]

| Primer and purpose | sequence |
| --- | --- |
| **For 5’RACE** |  |
| A-GSP1 | TCTGGCCGCAGCCGTTCCAT |
| A-GSP2 | TGGCCGCAGCCGTTCCATC |
| A-GSP3 | ATCACCCCTTCCACCGAGAT |
| B-GSP1 | CGGTTCAAGTCGGCGAACATC |
| B-GSP2 | TCAAGTCGGCGAACATCTGCT |
| B-GSP3 | TCCTTCAGCGCCTGCTCGAT |
| **For construction of pJTU3700** |  |
| 3700F | GAATCTAGAGCAGCCAAGCTTATCGA |
| 3700R | GAATCTAGATCAGGTCAGCACGGTCATGAA |
| **For construction of pJTU3707** |  |
| PdndBF | GCCGGATCCAGGACCGCCTTGTGCTCGATG |
| PdndBR | GACGGATCCTTGATCAGGAATCGGGCAGCC |
| **For deletion of -10 region of *dndB* promoter** | |
| B10F | TCGTACGTCAGGTAAATGACGTACGG |
| B10R | GAGCGGGTGGGATCTTCTGCA |
| **For semiquantitative real-time PCR** | |
| RTdndBF | GAGTACGTTTTTCCGGCCATCC |
| RTdndBR | TCCTTCAGCGCCTGCTCGAT |
| **For quantitative real-time PCR** | |
| rrnAF | AGTAACACGTGGGCAACTGC |
| rrnAR | CTCAGACCAGTGTGGCCGGT |
| QdndBF | GATGGCTGCCCGATTCCT |
| QdndBR | GGTTCAAGTCGGCGAACAT |
| Q15855F | CTCGCCCTGCTTGCCGATGC |
| Q15855R | CGGGTACGCGGCCTGTTT |
| Q12925F | TCCTTGGGCGGGTTGACGA |
| Q12925R | CCATCCAGCAGTCCGAAGTCC |
| Q32700F | CACCAAGCTCACCACCTCCG |
| Q32700R | GGCAGTTCTTCTTGGCGTCC |
| Primer and purpose | sequence |
| Q25315F | CGACACTGCGAAGGACCACA |
| Q25315R | GACCACGAGTACGGCACCCT |
| Q25320F | TCGCCGGAGAAGCCGAGGAT |
| Q25320R | ACGAGGGTCAGTGGAAGGTC |
| Q01475F | CGGCACTTTCACGGTCACTC |
| Q01475R | AGGTCCCAGTTGCCCTCGTC |
| Q02375F | GTCCTGCCAGATCAGGGTCTCC |
| Q02375R | TCTCCCGCCGGTTCTACGA |
| Q02905F | GCTGACCCTGAACCGCAACC |
| Q02905R | TCTGCGTGTCCAGGTACGAGAA |
| Q31365F | CTGCGAGTGGATGAAGTCCG |
| Q31365R | TGAAGTTCTACACCGAGGAGGG |
| Q37155F | GTGGGAGGTGTAGCCGTTCA |
| Q37155R | CGACATGCAGTGGGACTTCTGG |
| **For construction of sigR mutant in Streptomyces lividans 1326** | |
| 5505LF | GAATCTAGACGCCCGGATGATCAACGCC |
| 5505LR | GACAGATCTGGTCGACTCCGCGCCCGTA |
| 5505RF | GAAAGATCTCACCGTGGACGCCGTCAGC |
| 5505RR | GCAGAATTCCCGTTCGTGGTGGTGCAGG |
| 773F | GCAAGATCTATTCCGGGGATCCGTCGACC |
| 773R | GCAAGATCTTGTAGGCTGGAGCTGCTTC |
| 5505TF | CACTGGGACCGACGCAGGGAC |
| 5505TR | CCGAGCCTTTCGCTTCGTTCG |
